# Unstable Prefrontal Response to Emotional Conflict and Activation of Lower Limbic Structures and Brainstem in Remitted Panic Disorder

**DOI:** 10.1371/journal.pone.0005537

**Published:** 2009-05-20

**Authors:** Natalya Chechko, Renate Wehrle, Angelika Erhardt, Florian Holsboer, Michael Czisch, Philipp G. Sämann

**Affiliations:** Max Planck Institute of Psychiatry, Munich, Germany; James Cook University, Australia

## Abstract

**Background:**

The neural mechanisms of panic disorder (PD) are only incompletely understood. Higher sensitivity of patients to unspecific fear cues and similarities to conditioned fear suggest involvement of lower limbic and brainstem structures. We investigated if emotion perception is altered in remitted PD as a trait feature.

**Methodology/Principal Findings:**

We used blood oxygenation level-dependent (BOLD) functional magnetic resonance imaging (fMRI) to study neural and behavioural responses of 18 remitted PD patients and 18 healthy subjects to the emotional conflict paradigm that is based on the presentation of emotionally congruent and incongruent face/word pairs. We observed that patients showed stronger behavioural interference and lower adaptation to interference conflict. Overall performance in patients was slower but not less accurate. In the context of preceding congruence, stronger dorsal anterior cingulate cortex (dACC) activation during conflict detection was found in patients. In the context of preceding incongruence, controls expanded dACC activity and succeeded in reducing behavioural interference. In contrast, patients demonstrated a dropout of dACC and dorsomedial prefrontal cortex (dmPFC) recruitment but activation of the lower limbic areas (including right amygdala) and brainstem.

**Conclusions/Significance:**

This study provides evidence that stimulus order in the presentation of emotional stimuli has a markedly larger influence on the brain's response in remitted PD than in controls, leading to abnormal responses of the dACC/dmPFC and lower limbic structures (including the amygdala) and brainstem. Processing of non-panic related emotional stimuli is disturbed in PD patients despite clinical remission.

## Introduction

Panic disorder (PD) is characterized by inexplicable bursts of severe anxiety and catastrophic cognition accompanied by cardiorespiratory and other physical sensations [1]. Agoraphobia and other types of phobic avoidance may be present from the start or develop during the disease course along with anticipation anxiety, adding to the debilitating psychosocial sequelae of PD [1], [2]. In addition, PD is characterized by high comorbidity with other anxiety disorders, major depressive disorder and bipolar disorder [1]. Selective serotonin (5-HT)-reuptake-inhibitors (SSRIs) prevent spontaneous panic attacks and are recommended for pharmacotherapy in PD[3].

Strong autonomous reactions during panic attacks have led preclinical studies to focus on the anatomical network that mediates endocrine, autonomous and behavioural reactions during acute fear [4]. This fear network encompasses the amygdala, thalamic and brainstem nuclei, medial hypothalamus, hippocampus and cortical areas such as the cingulate, medial prefrontal cortex (PFC) and insula[4], [5]. The amygdala plays a key role in this neural model of fear as it evaluates incoming sensory stimuli with regard to potential threat [4], [6]. Right amygdala activation was detected during a spontaneous panic attack in a patient with PD [7] and increased metabolism in the bilateral amygdala, hippocampus, and thalamus, midbrain, caudal pons, medulla, and cerebellum was reported for PD patients[8]. Amygdala volume reductions [9], volume reduction in the right dorsal ACC [10], volume increases of the brainstem [11] and the left insula, the midbrain and the pons [12] in PD have added to the notion that structures of this network are involved in the pathophysiology of PD. Likewise, there is evidence from neuroimaging studies that the brain's response during fear conditioning shows anatomical overlap with the response to emotional paradigms in anxiety disorders including social anxiety, specific phobia and posttraumatic stress disorder, with a predominant role of the amygdala and insula [13].

Many of the symptoms experienced by patients with PD, including persistent concerns about future panic attacks or worries about the implications of the attack such as losing control, having a heart attack, or ‘going crazy’, suggest that emotional processing may be affected in PD at a more general level [1]. Anticipation anxiety and phobic avoidance may even occur before panic attacks, underscoring that these symptoms are not merely consequences of previous panic attacks [2], [4]. As one potential source of altered emotional processing, neurocognitive models of PD have proposed information processing biases such as an attention shift towards fearful stimuli, with the result of physiological stimuli or otherwise non-fearful stimuli triggering overproportional anxiety [14]. Such attentional bias has indeed been detected in PD, e. g. by emotional Stroop analogues[15], [16], though negative results have been reported as well [17]. Larger distractibility by panic-related threat words was also accompanied by stronger limbic responses in PD patients compared with controls [18]. In a behavioural study, information processing bias markers proved to be predictors of panic symptoms including affective, behavioral, and cognitive symptom measures [19].

These interweaved observations in PD – attentional bias towards fear stimuli, acute eruptions of fear during panic attacks, and phobia and anticipatory anxiety – make more integrated neuroanatomical models of emotional processing an issue. In humans, the extent to which a stimulus is identified as emotive and linked to the production of an affective state, appears to depend upon the level of activity within two neural systems [20]: A ventral neural system including the amygdala, insula, ventral striatum, ventral ACC and prefrontal cortex (PFC) that supports the identification of the emotional significance of a stimulus, production of an affective state and the automatic regulation of emotional responses; and a dorsal neural system including the hippocampus, dorsal ACC and PFC that supports the regulation of affective states and subsequent behaviour [20]. There is substantial evidence from neuroimaging studies that these systems and their interplay are indeed involved in the expression fear and anxiety: for example, anxiety and harm avoidance traits can be predicted by the connection strength between the ACC and amygdala during emotion processing [21], [22]. Also, fear expression and conditioning, as well as anticipation of negative emotion, involve the participation of the dorsal ACC [23]–[25]. The medial PFC and ACC were further reported to mediate placebo induced anxiolytic effects [26] as well as the effortful regulation of affective states and subsequent behaviour [20].

While emotional processing as such has not been studied extensively in PD, anatomical regions reported as dysfunctional in PD and regions involved in related experiments such as panic anticipation or experimentally induced panic demonstrate overlap with the mentioned emotional processing networks: During panic attacks induced in healthy subjects by cholecystokinine-4, a strong panicogenic agent, activation of a wide range of networks including the orbitofrontal cortex, medial PFC and ACC [27], [28] was observed. Similarly, the anticipation of panic attacks activated a wide network spanning the ACC, hippocampus, orbitofrontal cortex and dorsolateral PFC (DPLFC) [29]. Contradictory results have been brought forward by studies in PD with regard to the response to facial affect: Increased cingulate response to neutral facial affect in PD patients [30], no differences in amygdala activation and greater ACC response to happy facial affect [31] and *reduced* responsivity to fearful faces in the ACC and amydala [30] have all been reported.

In this study we focussed on remitted PD patients, aiming at a characterization of emotional processing in the absence of acute PD symptoms. We employed a recently developed variant of a Stroop test referred to as emotional conflict paradigm [32]. Stroop tests are established instruments to study the response of the brain to competing information as represented by target and distractor stimuli [33]. Usually, behavioural interference (i. e. slowing during processing of semantically incongruent information) is observed as attention resources have to be allocated for the inhibition of the faster automatic responses (e. g. reading of words) in favour of the slower voluntary response (e. g. naming of colors) [34]. In typical emotional analogues of Stroop tests [15] the words are emotionally neutral or salient. As it is, those tasks mostly measure the ability of emotional stimuli to withdraw attention from the main task. In healthy subjects, however, they do not lead to robust behavioural interference [15], [32]. As recently suggested, the use of both emotionally salient target and distractor stimuli [32], [35] can overcome this limitation. Rather than distractibility from a cognitive task by emotional stimuli, the brain's specific response to competing emotional stimuli can be studied.

Against this background, we employed the emotional conflict paradigm [32] to explore whether the processing of emotional information, particularly incongruent information, is altered in patients with remitted PD. Moreover, on the basis of an event-related design, changing processing strategies of the brain depending on the stimulus order were explored [32], [35], [36].

## Materials and Methods

### Participants

Eighteen adults with remitted PD were recruited from the Anxiety Disorders Outpatient Clinic at the Max Planck Institute of Psychiatry, Munich. The diagnosis was ascertained by trained psychiatrists according to Diagnostic and Statistical Manual of Mental Disorders (DSM)-IV [37] criteria. At the time of inclusion and throughout the entire study period, all patients received monotherapy with a selective serotonin reuptake inhibitors (SSRI) with no changes in the dosage and no additional medication. All patients were remitted as defined by a Panic Symptom Severity Score below 7 [38]. Patients with anxiety disorders due to a medical or neurological condition, or with primary unipolar depression or bipolar disorder were not included.

The control group comprised 18 age and gender matched healthy subjects recruited by local advertisements. A computerized version of the Munich-Composite International Diagnostic Interview [39] was employed to exclude neuropsychiatric conditions, and a medical history was taken. Major medical illnesses, previous head injury with loss of consciousness and substance abuse or dependence were further exclusion criteria. All participants were right-handed and native speakers of German. The protocol was approved by the local ethics committee, and informed written consent collected from all participants. Demographic and illness-related variables are summarized in **Table 1**.

**Table 1:** Demographic and clinical characterization

	Patients	Controls	p-value^1^
N (female/male)	18 (10/8)	18 (10/8)	N/A
Age, years	34.4 (8.0)	30.0 (6.4)	n. s.
ICD-10 diagnosis, N (%)		N/A	
PD and agoraphobia (F40.01)	14 (77.8%)		
PD (F41.0)	4 (22.2%)		
Age at onset, years	23.0 (6.2)		
PAS^2^ at time of treatment initiation	30.5 (8.8)		
**Psychopathological assessment at time of fMRI**			
PAS^2^ at time of study entry	3.3 (1.7)	N/A	N/A
State anxiety score (STAI-X1)	37.2 (6.3)	30.2 (5.9)	0.002
Trait anxiety score (STAI-X2)	36.4 (6.8)	30.2 (5.3)	0.005
Beck Depression Inventory (BDI)	3.4 (3.3)	0.4 (1.0)	<0.0001
**Selective serotonine reuptake inhibitor treatment**			
Citalopram, N (%)	11 (61.1%)	N/A	
Dosage range	[20–40 mg]		
Escitalopram, N (%)	5 (27.8%)		
Dosage range	[10–30 mg]		
Paroxetine, N (%)	2 (11.1%)		
Dosage	[20 mg]		

Means and standard deviations are given unless stated otherwise.

1 Two-sided test for independent samples, significance threshold 0.05.

2 PAS: Panic Agoraphobia Score[38]

### Psychopathological assessment

All subjects received a clinical MRI protocol for screening purposes and to become acquainted with the MRI environment. State and trait anxiety and levels of depressive mood were assessed using self-rating questionnaires (STAI [40] and Beck Depression Index [41]) at study inclusion and before the experiment. PD symptom severity was assessed using the Panic and Agoraphobia Scale [38] at study inclusion. All subjects received a standardized instruction to the paradigm and a 5-minute training session outside the scanner.

### MRI data acquisition

Imaging was performed on a 1.5 Tesla MRI scanner (Signa Echospeed, 1.5 Tesla, General Electric, Milwaukee, Wisconsin, USA) using an 8-channel headcoil. For fMRI, T2*-weighted echoplanar images (EPI, single shot pulse sequence, TR/TE 2000 ms/TE 40 ms, flip angle 90°) were obtained parallel to the anterior/posterior commissure plane (25 slices, FOV 22×22 cm^2^, matrix 64×64, slice thickness 3 mm, 1 mm gap, resolution 3.44×3.44×4 mm^3^). The fMRI session comprised 365 images (TR 2 s). Last, a high resolution T1-weighted image was obtained.

### FMRI paradigm

**Figure 1:**
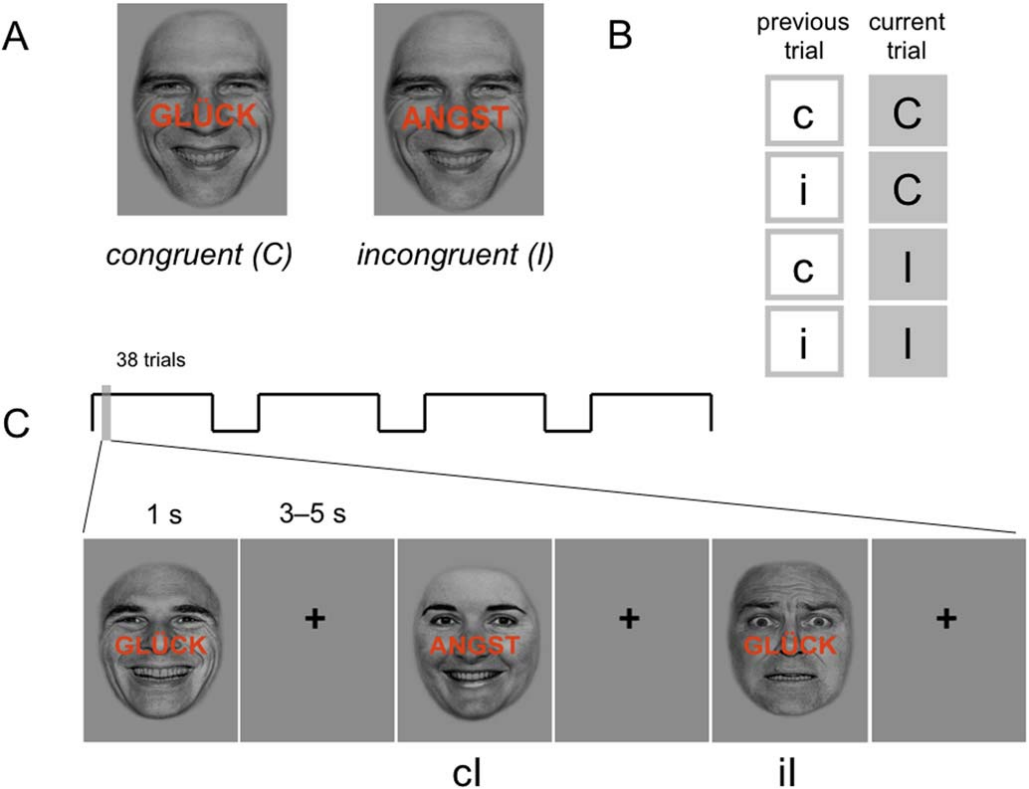
Emotional conflict paradigm. (A) Basic stimulus material consisting of congruent and incongruent face expression/word pairs from eight subjects of the Ekman faces collection (fearful and happy face expression; words ‘happy’ and ‘fear’)[42]. (B) Categorization of trials into four order types (cC, iC, cI, iI) depending on congruence of the previous and the current trial. (C) Each 38 randomized stimuli were presented in four blocks (see methods section) with jittered interstimulus interval. The second trial in the depicted example would be classified as ‘cI’, and the third trial as ‘iI’, also referred to as low and high conflict resolution trials [32].

We employed a German version of the emotional conflict paradigm as described by Etkin et al.[32] The paradigm is a variation of an emotional Stroop task with single trials being combinations of an emotional face in the background (happy or fearful expression) and the words ‘GLÜCK’ or ‘ANGST’ (German for ‘HAPPINESS’ and ‘FEAR’) printed across the face in bold capital red letters (**Figure 1**). Faces were taken from each four men and women of the original Ekman faces set [42], adjusted to an oval shape with standardized positions of the eyes and the mouth and normalised brightness[32], [43]. Trials were displayed for 1000 ms with a jittering interstimulus interval (4.00±0.38 s, range 3–5 s (Presentations software, Neurobehavioral Systems, Albany, USA) via a mirror attached to the head coil. One run consisted of 152 trials in sections of 38 trials and breaks of 30 s. Between face presentation, a fixation cross was shown. Depending on the congruence between face expression and word, trials were classified as congruent (C) or incongruent (I). Trials were encoded according to congruence of the previous trial, resulting in four order types: cC: congruent trial following a congruent trial; cI: incongruent trial following a congruent trial; iC: congruent trial following an incongruent trial; iI: incongruent trial following an incongruent trial. Order types were counterbalanced across the experiment. To avoid priming effects, direct repetitions of the same face and repetitions of the same face-word-distractor combination (e. g. happy face, word ‘fear’) were excluded [32], [44], [45]. Participants were instructed to identify the face expression and answer as quickly and precisely as possible by pressing the right (happy face) or left (fearful face) answer button with the index finger.

### Analysis of behavioural data

Reaction times (RTs) collected during the fMRI experiment were analyzed. Error trials (wrong answers, omissions and double responses), posterror trials and trials with outlier RTs (>three interquartile lengths below/above 1^st^/3^rd^ quartile) were excluded from any RT calculations. For accuracy calculations, all types of errors were considered. For later factorial analyses of variance, each trial was assigned to the factors face/word *combination* (4 levels), face type (*emotion*, 2 levels), word type (*word*, 2 levels), *congruence* of the current trial (2 levels), congruence of the previous trial (*previous*, 2 levels) and patient or control group status (*group*, 2 levels).

### FMRI analysis

#### Image preprocessing

Images were processed using Statistical Parametric Mapping (SPM) software (version SPM5, http://www.fil.ion.ucl.ac.uk/spm) on a Linux workstation. The first five images of each time series were excluded due to unequilibrated T1-effects. All remaining images were slice-time corrected and realigned to the first image. One subject was excluded due to excessive motion (translation or rotation beyond 2 mm or 0.1 degrees, respectively). Images were normalized to a standard EPI template (SPM5 distribution; linear and non-linear transformations, 5^th^ degree spline interpolation to 2×2×2 mm^3^ resolution) and smoothed (Gaussian kernel, full width half maximum 8×8×8 mm). Motion parameters were compared between patients and controls by submitting the maximum extent of translation (x-, y- and z-plane) or rotation (pitch, roll, yaw) coefficients gained from the realignment procedure as well as the root mean square movement for each of these measures[46] to multivariate analysis of variance.

#### Statistical analysis

For fMRI, first level models were defined for each subject within the general linear model framework of SPM5. For the four order types (cC, cI, iC and iI), stimulus onset times were defined as stick functions in four regressors (R_1–4_). Error and posterror trials were modelled as separate regressors (R_5–6_) to avoid influences from error detection processes. Equally, the first trial after 30 s of rest was separated out (R_7_). Further nuisance covariates were affine motion correction parameters (R_8–13_) and global signal of grey matter, white matter and CSF (R_14–16_). Time series were high-pass filtered (maximum wavelength 128 s), and R_1–7_ were convoluted with the canonical hemodynamic response function [47]. After model estimation, 1^st^ level t-contrast maps were generated as follows: A: Stroop interference (I>C, and opposite contrast). B and C: Stroop interference split by previous trial type (B: previous trial congruent [Stroop_CON_]: cI>cC; C: previous trial incongruent [Stroop_CON_]: iI>iC). D: Comparison between the two Stroop subtypes: Stroop_INC_>Stroop_CON_, and opposite contrast. E and F: Conflict monitoring and conflict resolution as defined by Etkin et al. [32], i. e. cI>iI (conflict monitoring) and iI>cI (conflict resolution). 2^nd^ level random effect analyses were performed separately for patients and controls and between groups using one and two sample t-tests. Statistical inference was drawn at the cluster level with a cluster-defining threshold of p_voxel_ of 0.01 for within-group and 0.05 for across-group comparisons. Clusters with family wise error, whole brain corrected cluster p-values [48] (significance threshold 0.01) are reported (see **Text S1** for details on anatomical assignment).

## Results

### Psychopathological assessment before fMRI

Patients showed higher measures of state and trait anxiety and depression compared with control subjects before the scanning procedure (**Table 1**), however with absolute values in normal ranges [49]. At the time of fMRI patients were remitted from PD with considerable lower PAS scores compared with the time of treatment initiation (4.0±2.1 vs. 30.5±8.8, p<0.001) [38].

### Behavioural performance

Patients showed a slower performance than controls (p<0.0001). This accounted for both congruent and incongruent trials, and for all order types (all p<0.0001). Both groups showed robust behavioural interference in incongruent trials (‘Stroop effect’), however, interference was stronger in patients both in absolute (52 ms vs. 35 ms) and relative terms (8.3% vs. 6.1%) (group×congruence, F = 4.933, p = 0.026). No group×order interaction was detected.

**Figure 2:**
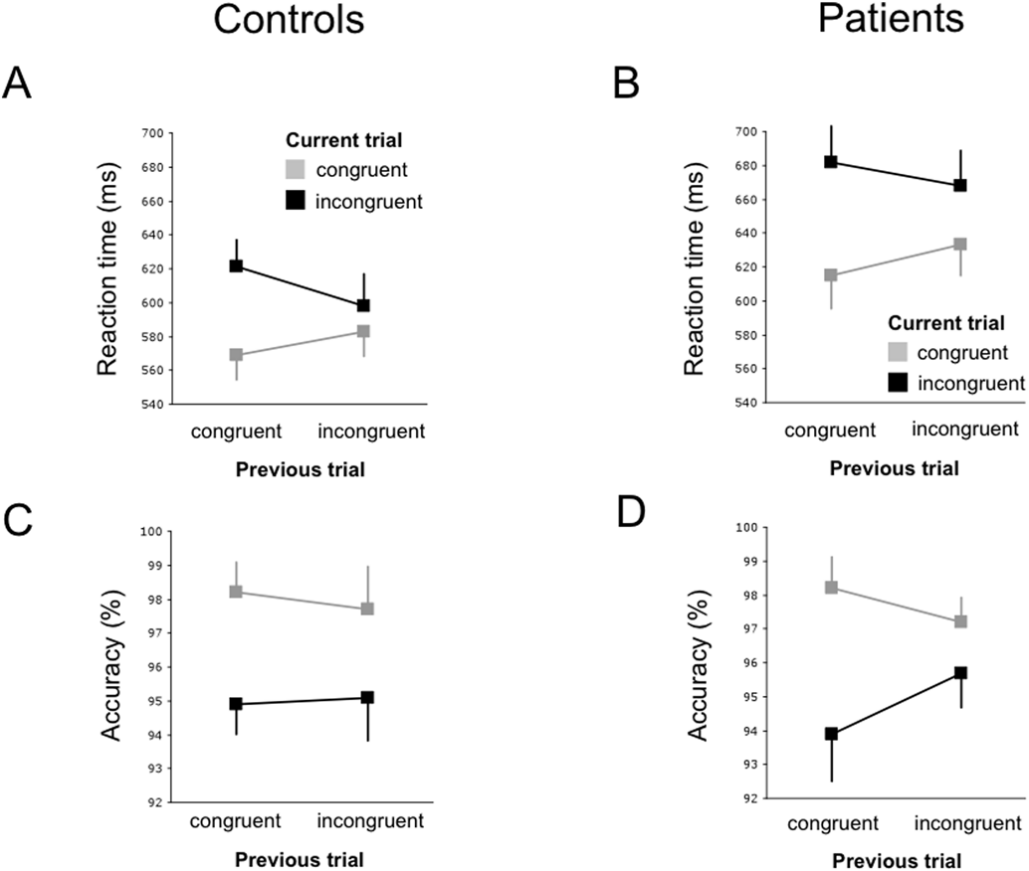
Conflict adaptation effects in controls and patients . Note generally slower RTs in patients. Labels on x-axes refer to the current trial. (A) For reaction times a significant previous×congruence interaction was found for both groups (controls: F = 13.760, p = 0.0002; patients: F = 8.104, p = 0.0045). (B) For accuracy rates an effect of congruence of the current trial (controls: F = 14.280, p = 0.003; patients: F = 7.328, p = 0.085) was found, but no effect of previous trial type or the previous×congruence interaction.

We then investigated the influence of preceding incongruence on the current trial (previous×congruence) as described for healthy subjects [32], finding that both groups showed this effect (controls: F = 13.760, p<0.001; patients: F = 8.104, p = 0.005, **Figure 2** ***a***) with no between-group difference (group×previous×congruence, F = 0.139, p = 0.709).

Three analyses were performed to disentangle the source of stronger interference in patients: (1) Interference was not different between groups for preceding congruence (group×congruence, p = 0.168) whereas for preceding incongruence patients showed stronger interference at trend level (group×congruence, p = 0.063). (2) RT acceleration of iI trials compared with cI trials due to successful conflict adaptation (referred to as Gratton effect) was found in controls (p = 0.008) but not in patients (p = 0.120; group×previous interaction, p = 0.500). (3) Slowing of congruent trials following incongruent trials was demonstrable in controls and patients (p = 0.009 and p = 0.010; group×previous interaction, p = 0.788). In neither group, RTs were different between face types or word types (p-values>0.132), or between the face/word combination constituting (in-)congruence between groups (group×combination, p = 0.173).

### Relationship between anxiety levels and performance

In patients, state anxiety levels were weakly correlated with mean RTs (state anxiety: rho = 0.496, p = 0.036). This association was also present for all congruent trials (rho = 0.529, p = 0.024), albeit weaker for incongruent trials (rho = 0.448, p = 0.068) and absent for the interference effect. Results were in similar range for trait anxiety scores. No associations were found in the control group.

### FMRI results

#### Effect of incongruence versus congruence in controls and patients

**Figure 3:**
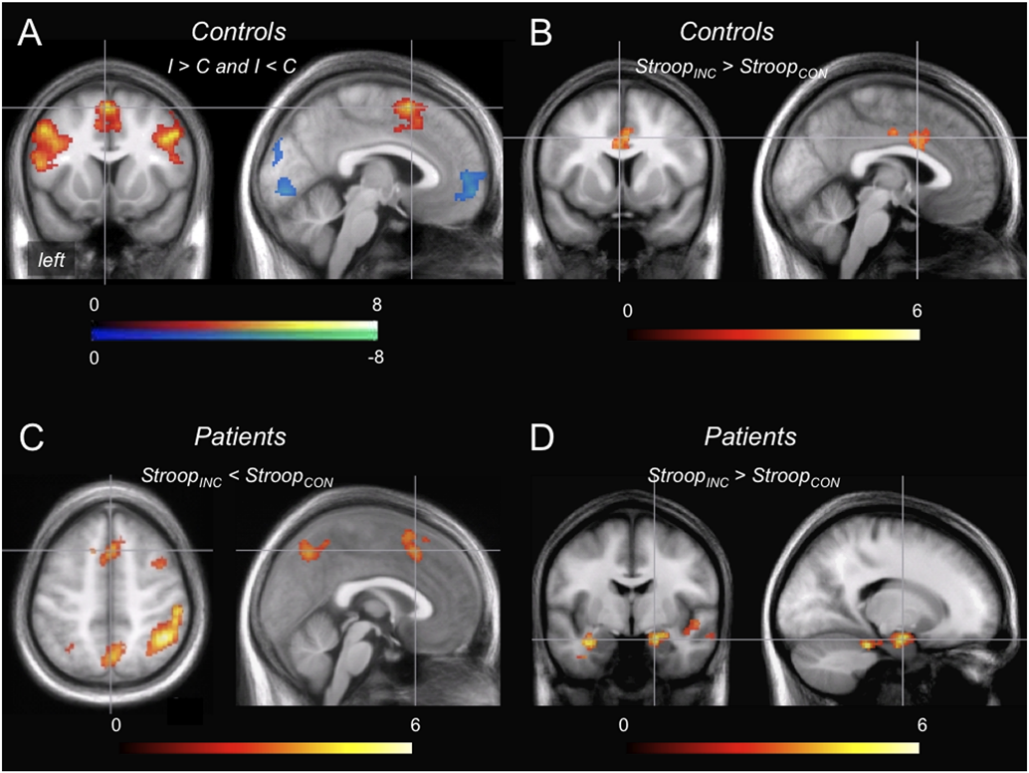
fMRI response to conflict in general and to conflict depending on the type of preceding trial as analyzed in the separate groups. (A) Significant clusters of activation and deactivation to incongruent vs. congruent trials (I>C) in controls (see supplemental data for result tabulation). (B) Control subjects showing stronger ventral ACC activation in conflict trials preceded by incongruent trials (Stroop_INC_>Stroop_CON_). (C) Contrary, patients showed more activation with preceding congruence in dACC/dmPFC (Stroop_CON_>Stroop_INC_) and further regions detailed in table 2. (D) In response to conflict trials preceded by incongruent trials, patients exhibited bilateral temporomesial including right amygdala, and brainstem activation (table 2).

In controls, the response to conflict (I>C) elicited BOLD activation in the left and right lateral PFC, dorsal ACC (dACC)/dorsomedial PFC (dmPFC) and a left parietal area (**Figure 3a** **, Table S1**). Deactivation was seen in regions compatible with default mode network [50]. Highly similar responses were seen for patients, with no differences in the 2^nd^ level comparisons to controls.

#### Consideration of preceding trial type within the control group

Splitting of the I>C comparison according to the preceding trial type revealed stronger ventral ACC recruitment for preceding incongruence (**Figure 3** ***b*** **,** **Table 2**). The opposite contrast – more activation with preceding congruent trials – revealed no significant results.

**Table 2:** Response to conflict depending on preceding trial type in patients and controls

Anatomical region	BA	k	FWE-corrected P_cluster_	Peak voxel	
				Z	x y z
*Controls: Increased response with preceding incongruent trials (Stroop_INC_>Stroop_CON_)*					
L/R ACC	L BA 24	446	0.006	3.43	−16 −4 34
	R BA 24, BA 33				
*Patients: Increased response with preceding incongruent trials (Stroop_INC_>Stroop_CON_)*					
L parahippocampal gyrus	Hippocampus, BA 35	1022	0.001	4.20	−34 −10 −22
L brainstem	Midbrain, pons				
R parahippocampal gyrus, amygdala	Amygdala^1^, BA 34, hippocampus, BA 28	542	0.001	4.39	22 −34 −26
R brainstem	Midbrain, pons				
R middle and superior temporal gyrus	BA 21^2^	448	0.005	4.52	48 −2 −26
*Patients: Increased response with preceding congruent trials (Stroop_CON_>Stroop_INC_)*					
R middle and superior frontal gyrus	BA 6, BA 8, BA 9	1330	0.001	3.85	34 8 60
R ACC	BA 32^3^				
R supramarginal gyrus, superior and inferior parietal lobule	BA 40, BA 2	1514	0.001	4.54	46 −50 46
R middle and superior frontal gyrus	BA 46, BA 9, BA 10	709	0.001	4.00	36 50 26
L/R precuneus	BA 7	457	0.001	3.85	10 −60 42

1 Coverage of amygdala: 63%.

2 Marginal extension to BA 22.

3 Marginal extension to left ACC (BA 32).

#### Consideration of preceding trial type within the patient group

The response to conflict depending on the preceding trial, however, was markedly different from controls: for preceding incongruence (Stroop_INC_), less activation was found in the dACC/dmPFC, right DLPFC and parietal areas compared with Stroop_CON_ while activation was stronger in the temporomesial cortices and the brainstem (**Figure 3** ***c–d*** **,** **Table 2**).

#### Between-group comparison of the Stroop_CON_ and Stroop_INC_ contrasts

**Figure 4:**
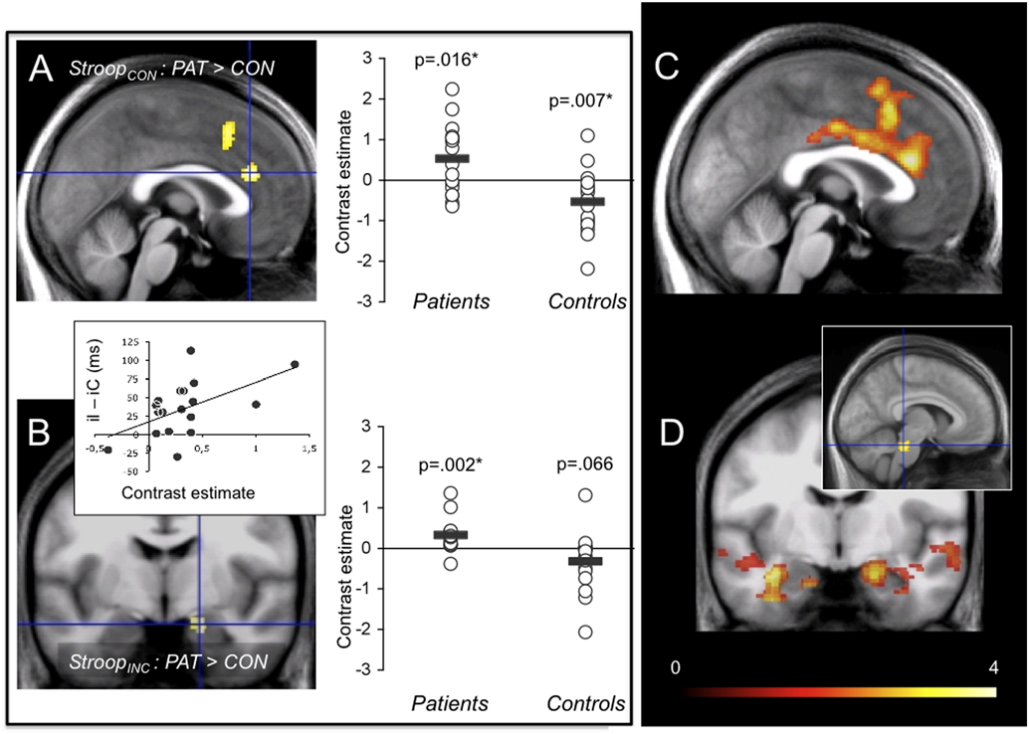
Across group comparison of response to conflict as separated by preceding trial type . (A) In response to conflict trials preceded by congruent trials (Stroop_CON_) patients showed stronger dACC/dmPFC activation than controls. This effect was based on activation in patients and deactivation in controls (contrast estimates extracted at peak voxel x = 2, y = 30, z = 22, cluster thresholded at p_voxel_<0.005). (B) In response to conflict trials preceded by incongruent trials (Stroop_INC_) patients showed stronger right temporomesial including amygdalar activation. Extraction of contrast estimates (peak voxel within amygdala mask, x = 18, y = −8, z = −20) demonstrated that activation occured only in patients, showing a positive correlation with their behavioural interference (rho = 0.461, p = 0.013). (C) Isolation of the effect of the previous trial type revealed less dmPFC/ACC activation in patients compared with controls when switching from incongruent to congruent background. (D) In turn, patients showed more activation of the bilateral amygdala-hippocampal complex and the brainstem. The inlay shows the most robust part of the cluster locating to the upper pons and midbrain area (p_voxel_<0.01, corrected p_cluster_ = 0.034).

To investigate if patients differed from controls already in the Stroop_CON_ condition, this contrast was taken to the 2^nd^ level, demonstrating more activation of the ventral ACC in patients. The extraction of contrast values demonstrated that the between-group difference was based on activation in patients and deactivation in controls (**Figure 4** ***a***). For preceding incongruence, patients showed more right temporomesial (including amygdala) and temporopolar activation than controls. Here, extraction of contrast values demonstrated significant activation only in patients (**Figure 4** ***b***). In addition, the patients' right amygdala response in the iI>iC contrast was positively correlated with the corresponding behavioural interference (**Figure 4** ***b***). Anatomical details of the activations are given in **Table S2**.

#### Isolation of the effect of previous trial type

The opposing effect of the type of preceding trial was isolated by the contrast Stroop_INC_>Stroop_CON_ taken to a 2^nd^ level comparison between groups. This confirmed that patients exhibited significantly less ACC and dmPFC engagement but more temporomesial, particularly right amygdala engagement when processing of incongruent material came under the influence of preceding incongruence (**Figure 4** ***c–d*** **,** **Table 3**). The effects of the previous trial type of congruent and incongruent stimuli were also analysed separately (**Text S2**, **Table S3**).

**Table 3:** Isolated effect of preceding trial type (Stroop_INC_>Stroop_CON_) compared across groups

Anatomical region	BA	k	FWE-corrected P_cluster_	Peak voxel	
				Z	x y z
*Patients>controls*					
R amygdala, parahippocampal gyrus	Amygdala^1^, hippocampus, BA 34, BA 35, BA 28, BA 27, BA 21, BA 22, BA 38	2768	0.001	3.78	16 6 −20
R middle and superior temporal gyrus					
L amygdala, parahippocampal gyrus	Hippocampus, amygdala, BA 34, BA 35, BA 28	2443	0.002	4.10	2 −28 −14
	BA 21, BA 47				
L middle temporal and inferior frontal gyrus					
L/R brainstem	Pons, midbrain				
*Patients<controls*					
L/R ACC	L BA 24, BA 32	3794	<0.001	3.72	0 28 20
	R BA 24, BA 32, L/R BA 33				
R superior frontal gyrus	BA 6, BA 8				
L/R posterior cingulate	BA 23				

1 Coverage of amygdala: 85%.

## Discussion

We investigated neural response patterns of remitted PD patients and control subjects to the emotional conflict paradigm, a Stroop-like presentation of incongruent and congruent emotional stimuli [32]. Our main observations were: 1. Patients reacted generally more slowly, showed stronger behavioural interference and less conflict adaptation. 2. FMRI was sensitive in unmasking differences of conflict adaptation. Here, patients demonstrated stronger dACC engagement than controls during conflict presentation in the context of preceding *congruence*. In the context of preceding *incongruence*, group differences intensified: while controls increased dACC activity and succeeded in reducing behavioural interference, patients lacked such further ACC recruitment and demonstrated right hippocampus/amygdala activation.

Stronger interference in PD has previously been reported for emotional/color-naming Stroop experiments using panic-threat words [18], [51] and general-threat words [51], and for the regular color-naming Stroop test [18]. These and similar studies [19], [52] integrate well with the hypothesis of an attention and information processing bias in PD. While such biases were found to predict clinical markers of PD [19], it is still a matter of debate whether they predate the onset of PD and represent vulnerability. Negative results in emotional Stroop tests in PD patients [17] and the absence of an attentional bias in healthy offspring of PD patients [53] suggest that these biases may develop throughout the disease. In addition to stronger interference to conflicting emotional stimuli we also observed generally slower performance in PD – irrespective of the trial type. This observation and the fact that stimuli were not panic-related suggests that processing of emotional stimuli may be altered at a more general level. Whether these alterations represent trait features of PD, however, needs to be addressed in different samples and by broader neuropsychological test batteries.

‘Potential confounds from SSRI treatment (or treatment×disease interactions) warrant further comment. In healty subjects, studies on effects of short oral citalopram were negative in most cases, reporting e. g. no effect on psychomotor performance [54] and working memory [55]. However, even a single intravenous citalopram dose was found to impact attention, categorization of facial affect, emotional memory and reactivity to threat in another study [56]. In patient samples, negative effects of SSRIs on memory functions have been reported, varying according to the underlying psychopathology [57]. Conversely, in patients with major depression, serotonin manipulation corrected attentional bias, face emotional recognition, emotional memory and decision making [58]. As a whole, the question whether longstanding SSRI treatment of the PD patients may have had an effect on their performance in this study, remains unclear.’

Another confound of the behavioural results may be anxiety *as* state anxiety has been reported to relate to interference induced by threat-related words in PD [59]. We found that patients' state anxiety levels were weakly correlated with RTs of all trials and of the congruent trials, but not with RTs of the incongruent trials and not with interference. Thus, stronger interference in PD with this paradigm appears to be more specific to PD than to concurrent anxiety.

In interference experiments, influences of the preceding on the current trial are a known phenomenon [60], [61]. More specifically, interference caused by an incongruent trial is lower if it follows an incongruent trial (iI) compared with an incongruent trial following a congruent trial (cI). Such adaptation is thought to be a consequence of conflict in the first incongruent trial leading to recruitment of supplementary cognitive control in the subsequent incongruent trial (iI) [60], [62]. This effect (referred to as Gratton effect) has been found in earlier cognitive Stroop experiments [60], [62]–[64], in the original report on the emotional conflict paradigm [32] and in our control group. Notably, patients lacked this effect. On the contrary, slowing in congruent trials following incongruent trials – allegeable by stronger attentional focus on the target stimulus that prevents response facilitation by the congruent distractor [65] – was equally strong in both groups. Overall, stronger interference in trials with preceding incongruent trials, relatively strongest slowing in iI trials, and absence of the Gratton effect argued for altered conflict adjustment in the patients.

In controls and patients, fMRI showed robust activation of the dACC/dmPFC and bilateral DLPFC and right parietal cortex in response to conflict. This pattern bears similarity to cognitive Stroop experiments, with dACC/dmPFC activation often attributed to conflict detection [60], [61], [65]–[68], and lateral prefrontal activation to execution of cognitive control [35], [61], [62], [65], [66], [69]. The ACC topography, however, was unexpected at first: As the paradigm is based on affective stimuli, activation of the more ventral (‘affective’) ACC division – as found in emotional Stroop analogues [16], [33] – rather than dorsal (‘cognitive’) ACC division was expected. However, in the emotional conflict paradim, there may actually be less demand to reconcile competing cognitive/emotional information streams [70] as conflict arises between emotionally salient stimuli. Assuming that no cognitive/emotional conflict is built up, our results are comparable to those of van der Heuven et al. who reported only minor group differences in a cognitive Stroop test [18]. Likewise, in line with our data, more dACC activation in tasks producing stronger interference[16] has been suggested. In the original report [32] the topography of the I>C contrast has not reported and can thus not be compared. A detailed discussion of results from the iI>cI contrast that was in the focus of the original report [32] is provided in the supporting information (**Text S3, Figure S1**).

The pivotal between-group differences emerged from analyses of effects of the preceding trial. Here, the brain's response to changing ‘background’ rather than the response to conflict itself is in the focus. Operationally, the effect of preceding incongruence was isolated by contrasting the I>C response of trials with preceding congruence (Stroop_CON_) to the I>C response of trials with preceding incongruence (Stroop_INC_). In controls the change from congruent to incongruent background was associated with an expansion of dACC activation (**Figure 3** ***b***) and a concurrent reduction of behavioural interference. In patients, the same analysis demonstrated *less* dACC and *more* engagment of the bilateral hippocampus and right amygdala. Our interpretation of this finding is that in PD interactions might exist between emotional conflict processing and the background (i. e. current state of the conflict processing networks) against which conflict processing takes place. The right amygdala response in patients was in addition positively correlated with the degree of behavioural interference, rendering the pattern rather dysfunctional. This concurs with a study showing that reactions of PD patients in an emotional Stroop with panic-related words were slower and associated with right amygdala/hippocampus activation [18]. It must be added, however, that there remains a gap between activation of fear network components at the fMRI level, and clinical panic attacks that cannot be easily bridged. Neither did panic attacks occur in patients of this study, nor were more subtle anxiety or endocrine/autonomous functions recorded to prove peripheral effects. Still, activation of the amygdala has been observed during spontaneous and CCK-4 induced panic attacks in healthy subjects [71], and amygdala activation predicted panic experienced by healthy subjects receiving CCK-4 [28].

Between-group comparison of the Stroop_CON_ (cI>cC) contrast allowed for a better understanding of the patients' response in that it demonstrated stronger (more ventral) ACC activation in conflict processing during congruent background. Such activation has been observed during anticipation of panic [28], [29], and similarly, during anticipation of anxiety in healthy subjects [24], [72], [73], leading to attenuation of anxiety and autonomous arousal [73]. In fact, patients showed concurrent *less* activation of lower limbic structures (**Table S2**), pointing to suppression of limbic activity that occurs primarily but may reverse under certain conditions.

The ACC reactions of patients – more engagement in conflict processing during congruent, but attenuated recruitment in the presence of incongruent background –can be be interpreted as (1) shift of baseline ACC involvement in emotional conflict detection, and (2), failure of additional ACC/dmPFC recruitment along with activation of limbic structures when conflict processing comes under the additional influence of an incongruent background. Both conclusions are based on the combined application of within-group and between-group analyses.

From a more general perspective, these results suggest that dysfunctional prefrontal responses could play a role in PD, as anticipated [74], and relate to activation of structures of the fear network. It may be hypothesized that there is an initially (or tonically) increased conflict monitoring/generation effort of the ACC that is not appropriately adjusted to further demands. An even wider extension of dACC/dmPFC abnormalities emerged when we isolated the between-group effect of a change of the background (**Figure 4** ***c***). Similarity of this distribution with reported postsynaptic 5-HT_1A_ receptor deficient areas revealed in symptomatic PD patients[75] and with recently reported dACC cortex volume deficits[10] was noted. The opposite contrast further revealed activation of a pontine/midbrain area (**Figure 4** ***d***) that incorporated the upper raphe nuclei. This area and the hippocampus have been shown to exhibit persistently reduced presynaptic 5-HT_1A_ receptor density in remitted PD patients, a finding interpreted as potential trait marker [76].

Our study is limited by the restriction to remitted, medicated PD patients. In healthy subjects, acute [77] and chronic SSRI adminstration [78] can attenuate limbic activation to emotional stimuli, and changes of prefrontal and paralimbic responses were observed in general anxiety disorder [79]. Therefore, in addition to treatment×disease interaction effects on BOLD and behavioural results cannot be excluded. Also, exclusive specificity of our results to PD cannot be claimed as no other anxiety disorder was investigated. Last, we can not refute that due to an increased risk of developing depression, given the diagnosis of an anxiety disorder, risk traits for depression have also influenced our results.

In summary, we investigated fMRI BOLD responses and behavioural responses during the processing of non-panic related congruent and incongruent emotional stimuli in remitted PD patients and healthy controls. Stimulus order had a markedly larger influence on the brain's response in the patient group, leading to abnormal responses of the dACC/dmPFC and lower limbic structures (including the amygdala) and brainstem. Our findings provide evidence that the processing of emotional stimuli is disturbed in PD patients despite clinical remission.

## Supporting Information

Figure S1 High vs. low conflict resolution contrast compared with incongruent vs. congruent contrast and default mode network deactivation. (A) Rostral ACC/frontomesial cortex activation in high resolution>low conflict resolution trials (iI>cI, all study subjects N = 36, 2 runs, p_cluster_<0.005 uncorrected). Note also posterior cingulate activation. (B) A similar region shows deactivation in incongruent trials compared with congruent trials (I<C, p_cluster_<0.001), as reported in detail for the control group in table 3. (C) BOLD signal decreases in areas compatible with the default mode network in response to trials of the emotional conflict paradigm (all trials pooled regardless of congruence or order characteristics, red: BOLD increase, blue: BOLD decrease). (D) Right DLPFC and dorsal ACC activation in low>high conflict resolution trials (p_cluster_<0.005 uncorrected).
(3.00 MB TIF)

Table S1 Effect of conflict (I>C) in controls
(0.04 MB DOC)

Table S2 Between-group comparison of response to conflict separated by previous trial type
(0.04 MB DOC)

Table S3 Effect of previous congruence on processing of congruent trials within and between groups (cC vs. iC contrast)
(0.04 MB DOC)

Text S1 Details on anatomical assignment of result clusters
(0.03 MB DOC)

Text S2 Effect of previous trial type on processing of congruent or incongruent trials
(0.03 MB DOC)

Text S3 Comparison of high vs. low conflict resolution trials
(0.03 MB DOC)
