# Supplementary figures and images for: Unstable Prefrontal Response to Emotional Conflict and Activation of Lower Limbic Structures and Brainstem in Remitted Panic Disorder
Source: PLoS One. 2009 May 20;4(5):e5537. doi: 10.1371/journal.pone.0005537 (PMC2680057; doi:10.1371/journal.pone.0005537)

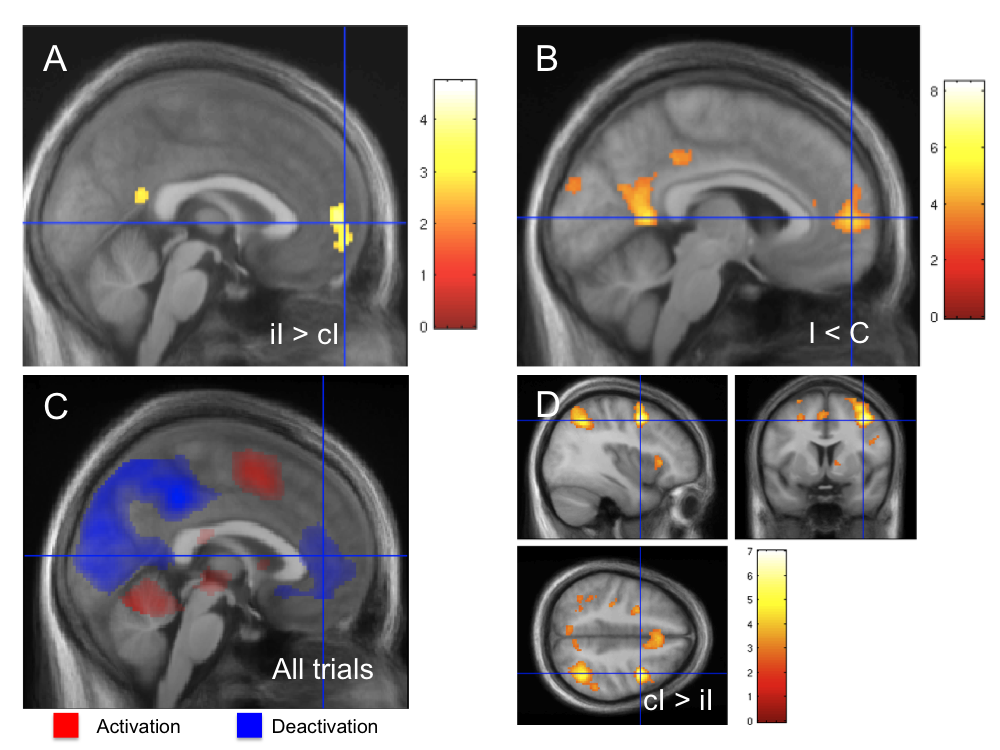

Supplement: Figure S1 — High vs. low conflict resolution contrast compared with incongruent vs. congruent contrast and default mode network deactivation. (A) Rostral ACC/frontomesial cortex activation in high resolution>low conflict resolution trials (iI>cI, all study subjects N = 36, 2 runs, pcluster<0.005 uncorrected). Note also posterior cingulate activation. (B) A similar region shows deactivation in incongruent trials compared with congruent trials (I<C, pcluster<0.001), as reported in detail for the control group in table 3. (C) BOLD signal decreases in areas compatible with the default mode network in response to trials of the emotional conflict paradigm (all trials pooled regardless of congruence or order characteristics, red: BOLD increase, blue: BOLD decrease). (D) Right DLPFC and dorsal ACC activation in low>high conflict resolution trials (pcluster<0.005 uncorrected). (3.00 MB TIF) [file pone.0005537.s001.tif]
