# Supplementary material for: Unstable Prefrontal Response to Emotional Conflict and Activation of Lower Limbic Structures and Brainstem in Remitted Panic Disorder
Source: PLoS One. 2009 May 20;4(5):e5537. doi: 10.1371/journal.pone.0005537 (PMC2680057; doi:10.1371/journal.pone.0005537)
Supplement: Table S1 — Effect of conflict (I>C) in controls (0.04 MB DOC) [file pone.0005537.s002.doc]

**Table S1. Effect of conflict (I >** C) in controls

| Anatomical region | Brodmann areas | *k* | FWE-corrected  P_cluster_ | Peak voxel | |
| --- | --- | --- | --- | --- | --- |
|  |  |  |  | Z | x y z |
| *Positive BOLD response* | | | | | |
| L middle, inferior and precentral gyrus | BA 44, BA 9, BA 6, BA 45 | 2062 | <0.001 | 4.46 | -46 6 38 |
| R precentral, inferior, middle and superior frontal gyrus | BA 9, BA 46, BA 44, BA 6 | 1623 | <0.001 | 5.12 | 38 2 34 |
| L/R medial and superior frontal gyrus, ACC | BA 32, BA 6 | 980 | <0.001 | 4.34 | -2 8 56 |
| L superior and inferior parietal lobule | BA 7, BA 40 | 975 | <.001 | 4.37 | -24 -56 44 |
| *Negative BOLD response* | | | | | |
| L/R fusiform gyrus and cuneus | BA 17, BA 18, BA19 | 2216 | <0.001 | 4.37 | -14 -78 -6 |
| L/R medial frontal gyrus and ACC | BA 10, BA 32 | 769 | <0.001 | 4.20 | -10 48 -8 |
| L ACC | BA 24, BA 32 | 404 | 0.008 | 4.02 | -14 42 14 |
| L posterior cingulate, precuneus | BA 29, BA 30 | 388 | .010 | 4.10 | -8 -58 2 |

Notes: L and R denote left and right; BA, Brodmann area; FWE, family wise error; *k* refers to cluster size.

Peak voxel coordinates refer to Montreal Neurological Institute (MNI) space.
