# Supplementary material for: Unstable Prefrontal Response to Emotional Conflict and Activation of Lower Limbic Structures and Brainstem in Remitted Panic Disorder
Source: PLoS One. 2009 May 20;4(5):e5537. doi: 10.1371/journal.pone.0005537 (PMC2680057; doi:10.1371/journal.pone.0005537)
Supplement: Table S2 — Between-group comparison of response to conflict separated by previous trial type (0.04 MB DOC) [file pone.0005537.s003.doc]

**Table S2. Between-group comparison of response to conflict separated by previous trial type**

| Anatomical region | BA | *k* | FWE-corrected  P_cluster_ | Peak voxel | |
| --- | --- | --- | --- | --- | --- |
|  |  |  |  | Z | x y z |
| *Stroop_CON_: Patients > controls* | | | | | |
| L/R ACC | L BA 32, BA 24  R BA 32, BA 24, BA 33 | 2041 | 0.005 | 3.62 | 2 30 22 |
| *Stroop_CON_: Controls > patients* | | | | | |
| R amygdala, parahippo­campal gyrus  L/R subgenual ACC  L/R basal ganglia  L brainstem | Amygdala, BA 34, BA 35  L BA 25, R BA 25  L thalamus, putamen, caudate  R caudate, putamen  midbrain, pons | 2736 | 0.001 | 3.42 | -8 -36 -24 |
| *Stroop_INC_: Patients > controls* | | | | | |
| R amygdala, para­hippocampal gyrus  R superior and middle temporal gyrus  R basal ganglia | Amygdala, hippocampus, BA 34, BA 35, BA 36, BA 27, BA 28  BA 38, BA 21, BA 22  Thalamus, putamen | 2690 | <0.001 | 3.27 | 56 -6 -8 |
| *Stroop_INC_: Controls > patients* | | | | | |
| L/R ACC  L/R posterior cingulate, L precuneus | BA 24, BA 33  L BA 23, R BA 23 | 2552 | 0.001 | 3.95 | -14 44 24 |

Notes: L and R denote left and right; BA, Brodmann area; FWE, family wise error; *k* refers to cluster size.

Peak voxel coordinates refer to Montreal Neurological Institute (MNI) space.
