# Supplementary material for: Unstable Prefrontal Response to Emotional Conflict and Activation of Lower Limbic Structures and Brainstem in Remitted Panic Disorder
Source: PLoS One. 2009 May 20;4(5):e5537. doi: 10.1371/journal.pone.0005537 (PMC2680057; doi:10.1371/journal.pone.0005537)
Supplement: Table S3 — Effect of previous congruence on processing of congruent trials within and between groups (cC vs. iC contrast) (0.04 MB DOC) [file pone.0005537.s004.doc]

**Table S3. Effect of previous congruence on processing of congruent trials within and between groups (cC vs. iC contrast)**

| Anatomical region | BA | *k* | FWE-corrected  P_cluster_ | Peak voxel | |
| --- | --- | --- | --- | --- | --- |
|  |  |  |  | Z | x y z |
| *Control group cC < iC* | | | | | |
| L postcentral and precentral gyrus  L inferior parietale lobule | BA 2, BA 3, BA 4  BA 40 | 529 | <0.001 | 3.98 | -28 -24 58 |
| *Patients: cC > iC* | | | | | |
| L parahippocampal gyrus, amygdala | Hippocampus, amygdala, BA 34, BA 38 | 612 | <0.001 | 4.13 | -26 -12 -18 |
| R parahippocampal gyrus, amygdala | Amygdala, BA 28, BA 34 | 413 | 0.008 | 3.78 | 22 -6 -20 |
| *[cC > iC]_controls_ < [cC > iC]_patients_* | | | | | |
| R parahippocampal gyrus  R superior temporal gyrus | Amygdala, hippocampus, BA 28, BA 34, BA 35, BA 27  BA 38 | 2525 | 0.001 | 3.70 | 28 -34 -6 |
| L parahippocampal gyrus  L middle and superior temporal gyrus | Amygdala, hippocampus, BA 28, BA 34, BA 35  BA 38 | 2353 | 0.002 | 4.01 | -40 12 -20 |

Notes: L and R denote left and right; BA, Brodmann area; FWE, family wise error; *k* refers to cluster size.

Peak voxel coordinates refer to Montreal Neurological Institute (MNI) space.
