# Supplementary material for: Unstable Prefrontal Response to Emotional Conflict and Activation of Lower Limbic Structures and Brainstem in Remitted Panic Disorder
Source: PLoS One. 2009 May 20;4(5):e5537. doi: 10.1371/journal.pone.0005537 (PMC2680057; doi:10.1371/journal.pone.0005537)
Supplement: Text S1 — Details on anatomical assignment of result clusters (0.03 MB DOC) [file pone.0005537.s005.doc]

*Anatomical assignment of fMRI result clusters*

After visual cluster assessment by projection onto the T1 group average, the C. Holmes brain, and a Brodmann atlas in MNI space (http://www.sph.sc.edu/comd/ rorden/mricro.html), MNI coordinates of voxels of significant clusters were collected and assigned to macroscopic structures and Brodmann areas (BA) ([http://ric.uthscsa. edu/resources/talairach daemon](http://www.google.de/search?hl=de&client=firefox-a&channel=s&rls=org.mozilla:de:official&sa=X&oi=spell&resnum=0&ct=result&cd=1&q=http%3A//ric.uthscsa.edu/resources/talairach+daemon&spell=1)), and the percentage coverage of an anatomical region and Brodmann’s area by the cluster determined ([http://www.fil.ion.ucl. ac.uk/spm/ext/#MSU](http://www.fil.ion.ucl.ac.uk/spm/ext/#MSU)). Subregions are detailed if the cluster coverage exceeded 5% of the total anatomical volume, or 10% of the cluster volume.
