# Supplementary material for: Unstable Prefrontal Response to Emotional Conflict and Activation of Lower Limbic Structures and Brainstem in Remitted Panic Disorder
Source: PLoS One. 2009 May 20;4(5):e5537. doi: 10.1371/journal.pone.0005537 (PMC2680057; doi:10.1371/journal.pone.0005537)
Supplement: Text S2 — Effect of previous trial type on processing of congruent or incongruent trials (0.03 MB DOC) [file pone.0005537.s006.doc]

# *Effect of previous trial type on processing of congruent or incongruent trials*

Current incongruent trials: In the first presentation of the paradigm, the comparison of iI with cI trials allowed to detect active participation of the rostral ACC in conflict resolution (iI>cI) and right DLPFC activation during conflict monitoring (cI>iI) [1]. This pattern was not robustly detected in controls or patients in this study, however, emerged when all subjects (N=36) and data from two runs were pooled. Left premotor area (Brodmann areas 6 and 8, corrected p_cluster_<0.001) activation was detected in patients during conflict monitoring with across-group comparison not gaining significance.

Current congruent trials: Controls showed more activation in the left sensori­motor area in response to iC trials compared with cC trials. In contrary, patients showed bilateral hippocampal-amygdalar activation in the cC>iC contrast (within-patients: left: corrected p_cluster_<0.001; right: p_cluster_=0.008) also significant when compared between groups (left: corrected p_cluster_=0.002, right: p_cluster_=0.001; **Table S3**). No significant differences were seen in the respective opposite contrasts.

Reference List

1. Etkin A, Egner T, Peraza DM, Kandel ER, Hirsch J (2006) Resolving emotional conflict: a role for the rostral anterior cingulate cortex in modulating activity in the amygdala. Neuron 51: 871-882.
