# Supplementary material for: Unstable Prefrontal Response to Emotional Conflict and Activation of Lower Limbic Structures and Brainstem in Remitted Panic Disorder
Source: PLoS One. 2009 May 20;4(5):e5537. doi: 10.1371/journal.pone.0005537 (PMC2680057; doi:10.1371/journal.pone.0005537)
Supplement: Text S3 — Comparison of high vs. low conflict resolution trials (0.03 MB DOC) [file pone.0005537.s007.doc]

*Comparison of high vs. low conflict resolution trials*

Etkin et al. focussed on high vs. low conflict resolution trials (iI and cI) to distinguish conflict monitoring (cI>iI) from conflict resolution (iI>cI), finding midline dorsomedial PFC, bilateral DLPFC and amygdala activation during conflict monitoring, and rostral ACC activation during conflict resolution. The latter was characterized in depth and found to strengthen negative feedback connection to the amygdala [1]. In this study, the iI>cI contrast and its counterpart did not result in robust activation of these areas in patients or controls. A number of analyses were performed to clarify this contradiction: First, it was noted that pooling of all subjects and available runs allowed to detect the respective activations (**Figure S1*a/d***), suggesting lower power to discriminate between iI and cI trials due to methodological factors or sample specifity. Second, deactivation in a similar rostral ACC and frontomesial area (BA 10) was found for incongruent trials compared with congruent trials, and was found when suppression of the default mode network was visualized using all trials (**Figure S1*b/c***). Third, reaction times were faster in the healthy subjects of this study (mean 593 ms) compared to reported values (~850 ms) [1], and behavioural interference was weaker (35 ms vs. reported ~60 ms). As a whole, these results indicate that trial difficulty may play a role in the sense of a ‘trial ease’ explanation.

Reference List

1. Etkin A, Egner T, Peraza DM, Kandel ER, Hirsch J (2006) Resolving emotional conflict: a role for the rostral anterior cingulate cortex in modulating activity in the amygdala. Neuron 51: 871-882.
